# Protective effects of curcumin on epileptic rodent models by alleviating oxidative stress and inflammation: a meta-analysis and mechanism exploration

**DOI:** 10.3389/fphar.2025.1602716

**Published:** 2025-07-10

**Authors:** Peng Dai, Lingyu Xu, Peng Zhang, Zheng Liang, Yunhang Chu, Ziqiao Yu, Lin Cao, Peng Sun, Xia Li

**Affiliations:** ^1^ College of Traditional Chinese Medicine, Chang Chun University of Chinese Medicine, Changchun, China; ^2^ The Affiliated Hospital of Changchun University of Chinese Medicine, Changchun, China

**Keywords:** curcumin, epilepsy, oxidative stress, inflammation, meta-analysis

## Abstract

**Objective:**

The purpose of this study is to systematically evaluate the therapeutic effect of curcumin on rodent epilepsy models through a meta-analysis of multiple animal experiments. It will also explore its potential mechanism of anti-oxidative stress and anti-inflammation to provide a theoretical basis for the application of curcumin in the clinical treatment of epilepsy.

**Methods:**

A total of 23 eligible animal studies were identified by searching eight databases (up to March 2025), including PubMed, Embase, Web of Science, Cochrane Library, and CNKI, Wan Fang, VIP, CBM. SYRCLE’s risk of bias tool was used to assess the quality of the literature, and Meta-analysis was performed using Review Manager 5.4 and Stata 18 software. Primary outcome measures included epilepsy latency, Morris water maze escape latency, oxidative stress markers (MDA, GSH, SOD), inflammatory factors (IL-1β, TNF-α), and Glial fibrillary acidic protein (GFAP).

**Results:**

Meta-analysis showed that the curcumin intervention group significantly extended the epilepsy latency (SMD = 1.85, 95% CI = 1.05–2.64, P < 0.00001) and shortened the water maze escape latency (SMD = −1.69, 95% CI = −2.23–1.16, P < 0.00001). In terms of antioxidant indicators, curcumin significantly decreased MDA levels (SMD = −3.50, P < 0.00001) and increased GSH (SMD = 2.87, P < 0.00001) and SOD (SMD = 2.42, P < 0.00001). The anti-inflammatory results showed that the levels of IL-1β (SMD = −1.73, P = 0.04) and TNF-α (SMD = −1.65, P < 0.00001) were significantly decreased, and the levels of GFAP-positive cells were decreased (SMD = −1.72, P = 0.05). Subgroup analysis showed that medium and high doses (100–299 mg/kg and ≥300 mg/kg) of curcumin were more stable, but the low dose group (<100 mg/kg) did not conduct in-depth analysis due to insufficient sample size of individual indicators. Sensitivity analysis and funnel plots suggest robust results, but some publication bias exists.

**Conclusion:**

Curcumin can effectively improve epileptic seizures and cognitive dysfunction in epileptic rodents through the dual mechanisms of antioxidative stress (inhibiting lipid peroxidation and enhancing antioxidant enzyme activity) and anti-inflammatory (reducing the release of pro-inflammatory factors and inhibiting glial cell activation). However, species differences and potential publication bias have certain effects on the results, and high-quality clinical studies can be carried out in the future to verify their clinical application value.

## 1 Introduction

Epilepsy (EP) is a relatively common central nervous system disease at present, which is caused by abnormal discharges of neurons in the brain with high synchronization. According to incomplete statistics, as of 2019, there are more than 70 million patients worldwide (Thijs et al., 2019), with an annual incidence of 50.4∼81.7 per 100,000 people (Saxena and Li, 2017), and the survey finding that prevalence and incidence of epilepsy are becoming more and more common, especially for some low-income countries and regions, 75% of patients with active epilepsy have not undergone systematic treatment (Fiest et al., 2017; Falco-Walter, 2020). The impact of epilepsy is not only reflected in the patient’s brain damage. It also manifests in the psychological and physical pressures from society. These include the stigma of the illness, the discomfort during job applications, and the financial burden of long - term treatment.

At this stage, the treatment of epilepsy is still mainly based on oral antiepileptic drugs, which aim to stop seizures as early as possible without apparent side effects (Neligan et al., 2012). Of course, this treatment method also dramatically affects the quality of life of patients. Although this antiepileptic drug has been developed for the third generation of about 25 drugs, it has been statistically found to be only effective in about 66% of individuals in high-income countries (Devinsky et al., 2016), with a high recurrence rate and a short epilepsy latency.

With the development of antiepileptic drugs in treating epilepsy, more and more researchers are focusing on medicinal plants (Forouzanfar et al., 2021). Curcumin is a traditional Chinese medicine monomeric compound isolated from medicinal turmeric. In recent years, it has been shown to be more effective and safer and has been used to treat various chronic diseases, including neurodegenerative diseases, cardiovascular diseases, cancer, diabetes, etc. (Garodia et al., 2023). It has been reported that the active ingredient curcumin activates the expression of nuclear factor erythroid 2-related factor 2 (Nrf2) and its related genes, inhibits inflammatory factors, and alleviates neuroinflammatory response (Khayatan et al., 2022). There are also reports to explore the mechanism of active oxygen and oxidative stress, and the effects of astrocytes and microglia on epilepsy, and more and more experiments have confirmed the feasibility of curcumin in the treatment of epilepsy.

Meta-analysis refers primarily to a statistical method to collect the results of existing experiments and studies to assess treatment efficacy and clinical availability better (Yang et al., 2023). In order to explore the effect of curcumin on seizures and its therapeutic effect, we studied curcumin on rodent epilepsy models through meta-analysis, and verified whether the potential mechanisms of curcumin treatment are mostly antioxidant stress and anti-inflammatory.

This meta-analysis is registered with PROSPERO, the International Register of Prospective Systematic Reviews (Identifier: CRD420251010178).

## 2 Methods and materials

### 2.1 Search strategy

We searched English databases (including PubMed, Embase, Web of Science, and the Cochrane Library) and Chinese databases (including CNKI, Wan Fang Database, VIP, and CBM) for animal trials on the effects of curcumin on epilepsy. The search is current from inception to March 2025, and relevant search terms include a combination of “curcumin”, “epilepsy” (“rat,” “mouse,” or “rodent”). Literature collection also includes hand searching of citations that can be included in relevant documents, and the final collection of documents is included in Endnote21 document management software for management. For experiments that are missing some of the data we need in the article, we seek additional information from the article’s authors or extract the experimental data from the Origin Pro software. For study inclusion/exclusion, two reviewers independently screened articles. Discrepancies were resolved through consensus.

### 2.2 Inclusion criteria and outcome measures

Studies eligible for inclusion included: (1) original studies that were eligible for *in vivo* experiments in rodents; (2) the treatment disease was epilepsy, and (3) curcumin was used as the only intervention monotherapy, compared with a blank control or drug-loaded solvent; (4) The outcomes of the study included at least one of the following: seizure latency, Morris water maze escape latency, antioxidative stress-related indicators (including Super Oxide Dismutase [SOD], Glutathione [GSH], and Malondialdehyde [MDA]), and/or anti-inflammatory indicators (including Interleukin-1β [IL-1 β], Tumor Necrosis Factor-α [TNF-α]), and/or Glial fibrillary acidic protein (GFAP). We explicitly excluded studies involving non-rodent animals, interventions containing drugs other than curcumin, and those failing to report core outcome measures (e.g., latency, oxidative/inflammatory markers). If a study included multiple curcumin dosage groups, these were split into independent subgroups for analysis to ensure a one-to-one correspondence between the control group and each dosage group. We have omitted the detection method of the required inspection indicators. The measurement units of MDA, GSH, SOD, IL-1β, TNF-α, and GFAP in the index were unified, they were nmol/mg, umol/g, u/mg, ug/mg, pg/mg, and number of GFAP positive cells.

### 2.3 Data extraction

In the data extraction phase, we followed the Cochrane Handbook for Systematic Review of Interventions (Version 6.5, 2024) (Higgins et al., 2024), using two reviewers to extract data separately and draw a data extraction table that included: (1) the baseline content of the included studies (including the name of the first author, year of publication); (2) the characteristics of rodents, including rat or mouse, rat species and sample size; (3) agents to induce epilepsy models; (4) dosage of curcumin; (5) Outcome measures.

If the experimental data is not directly given in the article, we contacted the author first. If the data cannot be obtained, the experimental data were extracted using Origin Pro2024 software. By grabbing the histograms and line charts in the data as coordinate axes, and reproducing the graphics on Origin Pro, we can automatically generate the data size of each position on the coordinate axis. The data presented in the article using the mean ± standard error (SEM) format were uniformly converted to mean ± standard deviation (SD). In studies with multiple intervention groups, this analysis only considered data from the control and curcumin groups for epilepsy.

### 2.4 Quality assessment

Our reviewers assessed the quality risk of the included studies using SYRCLE’s animal study risk of bias tool (Hooijmans et al., 2014). The evaluation items included seven items: Selection bias, Performance bias, Detection bias, Attrition bias, Reporting bias, and Other bias, for a total of 10 questions. The included studies were scored by entering SYRCLE’s Animal Study Risk of Bias tool into Review Manager 5.4 software, and the scoring criteria were expressed as Low risks, Unclear risks, and High risks.

### 2.5 Statistical analysis

Our meta-analysis was performed using Review Manager 5.4 and Stata 18 software. Dichotomous or continuous data analyses were selected for outcome measures, standardized mean differences (SMD) and 95% confidence intervals (95% CI) were obtained, and forest plots were plotted. Heterogeneity was evaluated by I^2^ test and significance level, and the significance level was α = 0.1. An I^2^ > 50% or P < 0.1 indicated significant heterogeneity, for which a random effects model was used; otherwise, a fixed effects model was employed. If there was significant heterogeneity between studies, the cause of the heterogeneity was sought by removing each study to observe heterogeneity and Stata 18 software performing sensitivity analyses in turn. We performed subgroup analyses of data with high heterogeneity in the collected outcomes to see if there was a significant change in heterogeneity, and to investigate the effects of different doses on outcome measures (within 100 mg/kg, 100–299 mg/kg, and above 300 mg/kg). We also assessed publication bias by funnel plots and the Egger test. Egger’s regression was performed using Stata 18 software to test for publication bias, with a P-value <0.05 indicating the presence of bias. To investigate the effect of a study on the overall pooled effect size, we performed sensitivity analyses for these outcomes using Stata 18 software. In addition to heterogeneity, we judged the P < 0.05 for all outcomes statistically significant.

## 3 Results

### 3.1 Study selection and characteristics

**FIGURE 1:**
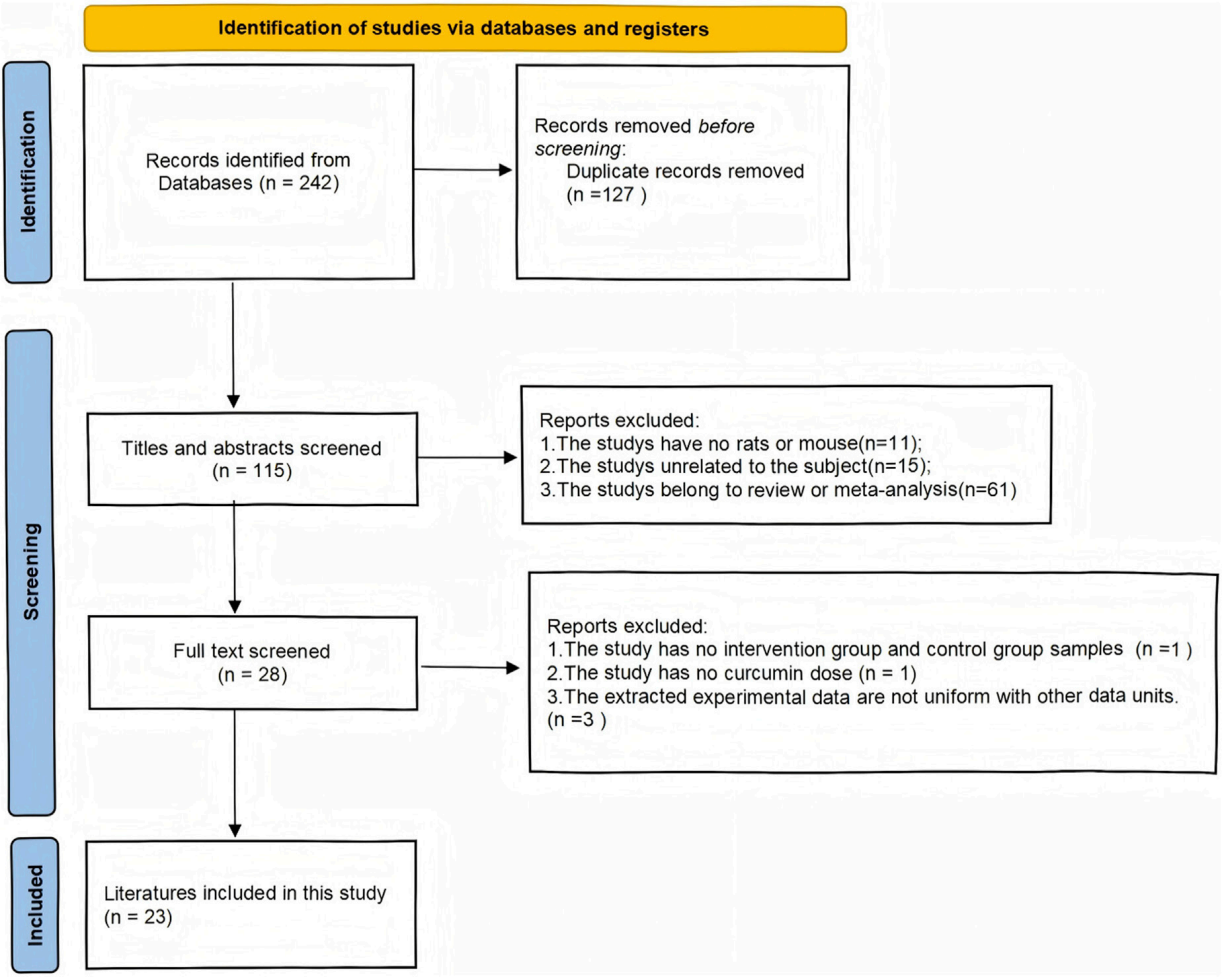
Flow diagram for systematic literature search.

As shown in (Figure 1) our search program identified a total of 242 articles: 23 from PubMed, 0 from the Cochrane Library, 38 from Embase, and 42 from Web of Science, for a total of 103 articles in the English database, leaving 47 after screening out duplicate articles. In the Chinese database: 45 articles from the CNKI, 32 articles from the Wan Fang database, 36 articles from the VIP database, 26 articles from the China Biomedical Literature Retrieval System (CBM), a total of 139 Chinese articles, and 68 duplicate literature. A total of 242 articles were published in Chinese and English, and 115 remained after deduplication. Among them, 11 articles were non-animal experiments, 15 articles were inconsistent with our meta-analysis, 61 articles were reviews or other meta-analysis literature, 1 article did not list the animal sample size of the intervention group and the control group, 1 article did not indicate the dose of curcumin, 3 articles did not have uniform outcome index units, and 23 articles were finally obtained.

Table 1 lists the underlying characteristics of the included studies. Nine studies were conducted in Sprague-Dawley (SD) rats (39.1%), 10 studies in Wistar rats (43.5%), one study did not specify the rat species (4.3%), and three studies were conducted in mice (13.1%). A total of 186 Wistar rats, 322 SD rats were treated with curcumin, 20 rats of unspecified species, and 92 mice were treated. The epilepsy models in the literature include Pentetrazol (PTZ) modelling, Pilocarpine modelling, Kainic acid (KA) solution modelling, and Fecl_3_ solution modelling. We divided the curcumin dosage in the literature into <100 mg/kg, 100–299 mg/kg, and ≥300 mg/kg, and there were 6, 14, and 8 articles (some articles related to multi-dose intervention) on antioxidant stress and anti-inflammatory indicators, respectively. 12 (52.2%) studies reported one or more biochemical indicators; Eleven studies (47.8%) did not report any of these indicators.

**TABLE 1:** Baseline characteristics of the included studies.

Number	Study ID	Cur dose (mg/kg)	Species	Sample size (cur/con)	Model	Outcomes mechanisms
1	Agarwal et al. (2011)	50/100/150	Healthy Swiss albino inbred mice	8:8	Ptz	GSH, MDA
2	Du et al. (2012)	30/100/300	SD Rat	15:15	Pilocarpine	Latency to seizure, GSH, MDA, SOD
3	Ezz et al. (2011)	80	Wistar Rat	10:10	Pilocarpine	GSH, MDA
4	He et al. (2018)	100	SD Rat	8:8	KA	MWM
5	Jahan et al. (2018)	150	Male mice	10:10	Ptz	Latency to seizure
6	Jiang et al. (2015)	100	Wistar Rat	6:6	KA	MWM, IL-1β, TNF-α, GFAP
7	Jyoti et al. (2009)	500/1,500	Wistar Rat	6:6	Fecl_3_	MWM
8	Kaur et al. (2014)	100	Wistar Rat	8:8	PTZ	GSH, MDA
9	Kaur et al. (2015)	100	Wistar Rat	6:6	PTZ	MWM, IL-1β, TNF-α, GFAP
10	Kiasalari et al. (2013)	100	Wistar Rat	14:14	KA	MDA, SOD
11	Kumar and Sharma (2020)	75	Wistar Rat	6:6	Fecl_3_	MWM
12	Mehla et al. (2010)	100/200/300	Wistar Rat	6:6	PTZ	Latency to seizure, GSH, MDA
13	Reeta et al. (2011)	300	Wistar Rat	6:6	PTZ	Latency to seizure, GSH, MDA
14	Saha et al. (2016)	100/200/300	Wistar Rat	8:8	PTZ	GSH, MDA
15	Slowing et al. (2023)	300	Rat	10:10	Pilocarpine	Latency to seizure
16	Huang et al. (2007)	300	SD Rat	30:30	Pilocarpine	Latency to seizure
17	Huang et al. (2006)	300	SD Rat	15:15	Pilocarpine	Latency to seizure
18	Xin (2009)	200	SD Rat	30:30	Pilocarpine	Latency to seizure
19	Liao and Mei (2017)	25/50/100	SD Rat	10:10	PTZ	MWM
20	Peng et al. (2007)	100	SD Rat	6:6	KA	Latency to seizure
21	Wu et al. (2022)	400	C57/BL6	20:20	KA	Latency to seizure, IL-1β, GFAP
22	Yan et al. (2011)	60	SD Rat	10:10	Pilocarpine	MWM, GSH, MDA, SOD
23	Yan (2017)	200	SD Rat	12:12	PTZ	Latency to seizure

Note: Abbreviations: SD, Sprague-Dawley; MWM, Morris water maze task; Cur, curcumin; GSH, glutathione; MDA, malondialdehyde; IL-1β, Interleukin-1β; TNF-α, Tumor Necrosis Factor-α; GFAP, glial fibrillary acidic protein.

### 3.2 Risk of bias

**FIGURE 2:**
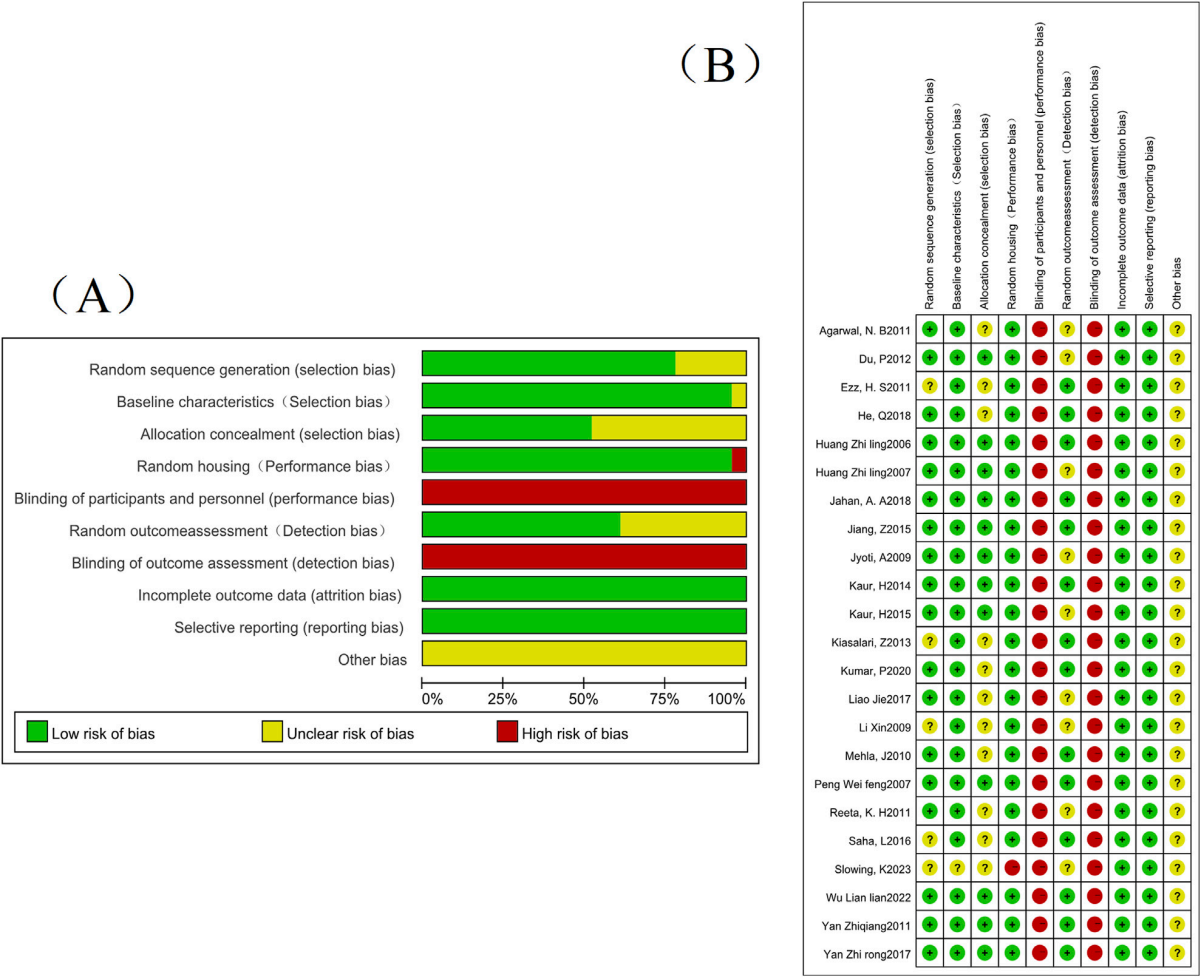
The risk of bias assessment of the 24 studies included in this meta-analysis based on SYRCLE’s risk of bias tool. Note: **(A)** Risk of bias graph; **(B)** Risk of bias summary.

About the results of the quality risk assessment, as shown in (Figure 2), 18 studies mentioned the words “random number table method” or mentioned random but did not describe the specific method, etc., and 12 studies described the random allocation method. Only one group of data collected in the article did not describe whether the baseline characteristics were similar or not; No article describes whether blinding was used; Only one set of experiments did not describe whether animals were randomly kept in captivity; Nine groups of studies did not describe whether animals were randomly selected for outcome assessment; None of the studies had incomplete or selective results; None of the studies were clear about other risks; All studies had complete data; Expected outcomes were reported in all studies.

### 3.3 Meta-analysis

#### 3.3.1 Epilepsy latency

**FIGURE 3:**
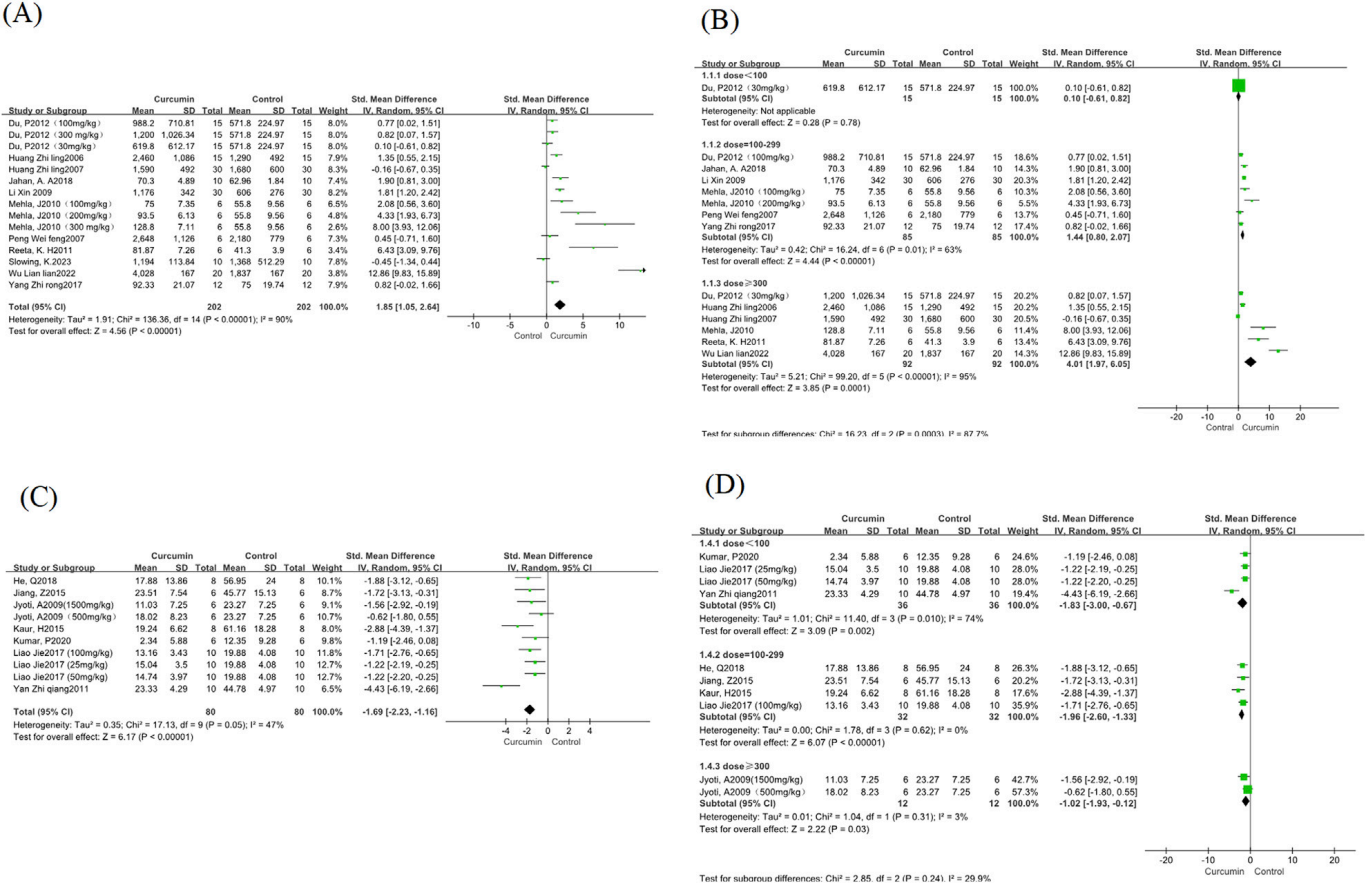
Epilepsy latency and Morris Water Maze escape latency forest map. Note: **(A)** Epilepsy latency forest map; **(B)** Epilepsy latency curcumin different dose subgroup forest map; **(C)** Morris Water Maze escape latency; **(D)** Morris Water Maze escape latency curcumin different dose subgroup forest map.

This study analyzed epilepsy latency in the 11 included studies. Standardized mean differences (SMD) and Hedges’ g method were used for outcome measures. There was significant heterogeneity between the articles (I^2^ = 90%, P < 0.00001), so a random-effects model was used for this indicator. The results showed that compared with the control group, the epilepsy latency in the curcumin treatment group was significantly increased (SMD = 1.85, 95% CI = 1.05∼2.64; P < 0.00001) (Figure 3A). In subgroup analyses of different curcumin doses, no significant differences were found between 100 and 299 mg/kg and ≥300 mg/kg curcumin doses (Figure 3B). However, it was observed that high-dose curcumin treatment had a better therapeutic effect on increased epilepsy latency. The low-dose group (<100 mg/kg) was not analyzed and interpreted because there was only one study.

#### 3.3.2 Morris Water Maze escape latency

The water maze experiment was analyzed for the escape latency of the water maze experiment in the 7 studies included in this study. Standardized mean differences (SMD) and Hedges’ g method were used for outcomes. There was a small heterogeneity between the articles (I^2^ = 47%, P = 0.05), so a random-effects model was used for this measure. The results showed that compared with the control group, the incubation period of curcumin treatment group was significantly reduced (SMD = −1.69, 95% CI = −2.23∼-1.16; P < 0.00001) (Figure 3C). According to the subgroup analysis of different curcumin doses, we found that the heterogeneity of the medium dose and high dose curcumin group was significantly reduced, and the low dose group had greater heterogeneity (Figure 3D), and the method of one by one elimination study found that after the exclusion of one study (Yan et al., 2011), the heterogeneity decreased significantly (I^2^ = 0, p = 1.0), and after reading through the full text, it was found that the reason for the heterogeneity may be due to the difference in the modeling agent, and the rats modeled with pirocarpine were more consistent with the characteristics of chronic spontaneous epilepsy, compared with PTZ and Fecl3 modeling, which is more likely to affect cognitive function in rodents (Lévesque et al., 2021).

#### 3.3.3 Antioxidant stress indicators

**FIGURE 4:**
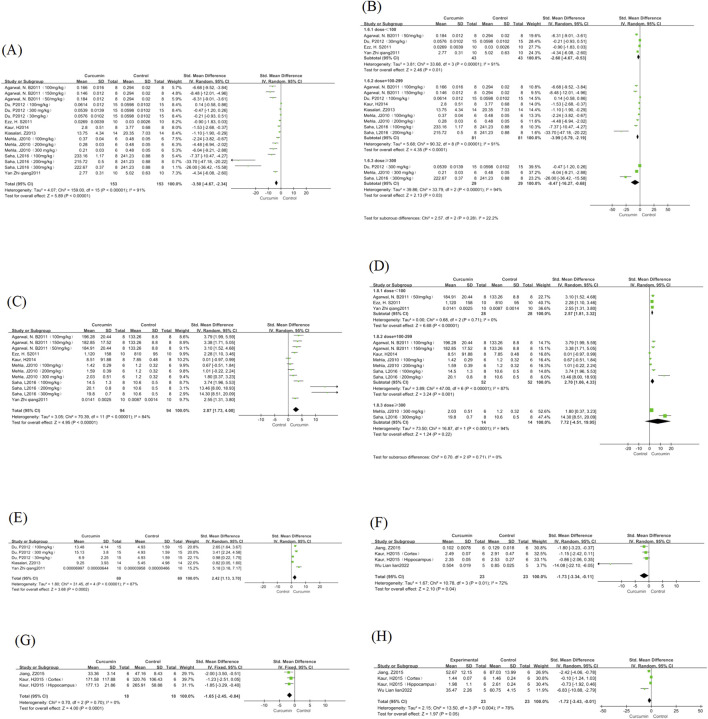
MDA, GSH, SOD, IL-1β, TNF-α, and GFAP forest map. Note: **(A)** MDA forest map; **(B)** MDA curcumin different dose subgroup forest map; **(C)** GSH forest map; **(D)** Subgroup forest map of different doses of GSH curcumin; **(E)** SOD forest map; **(F)** IL-1β forest map; **(G)** TNF-α forest map; **(H)** GFAP forest map.

This study involved the analysis of MDA content in the 8 included studies; Six studies involved the analysis of GSH content and three studies involved the analysis of SOD content. Standardized mean differences (SMD) and Hedges’ g method were used for outcomes. There was large heterogeneity between these articles, MDA (I^2^ = 91%, P < 0.00001); GSH (I^2^ = 84%, P < 0.00001); SOD (I^2^ = 87%, P < 0.00001), so a random-effects model was used. The results showed that compared with the control group, the MDA content in the curcumin treatment group was significantly reduced (SMD = −3.5, 95% CI = −2.23∼-1.16; P < 0.00001) (Figure 4A); The content of GSH increased significantly (SMD = 2.87, 95% CI = 1.73∼4.0; P < 0.00001) (Figure 4C), and the SOD content increased significantly (SMD = 2.42, 95% CI = 1.13∼3.70; P < 0.00001) (Figure 4E). We performed subgroup analyses for the MDA and GSH groups (Figures 4B,D), and the SOD group was not discussed due to the small number of studies in the literature. We found no significant change in the heterogeneity of the MDA group at different doses, and there was no significant difference in the reduction of MDA content between the three groups, considering that it may be related to the type of drug and the type of rat. The heterogeneity of the low-dose group was significantly reduced in the GSH group (I^2^ = 0%, p = 0.71). At the same time, there was no significant difference in heterogeneity between the medium-dose and high-dose groups, and the high-dose group showed that the data were meaningless.

#### 3.3.4 Anti-inflammatory indicators

This study involved the analysis of IL-1β content in the three included studies; Two studies involved the analysis of TNF-α content. There was heterogeneity in IL-1β (I^2^ = 72%, P = 0.01), and a random effect model was used. There was no heterogeneity in TNF-α (I^2^ = 0%, P = 0.7), and a fixed-effect model was used. Compared with the control group, the IL-1β content in the curcumin treatment group was significantly reduced (SMD = −1.73, 95% CI = −3.34∼-0.11; P = 0.04) (Figure 4F), TNF-α content was significantly reduced (SMD = −1.65, 95% CI = −2.45∼-0.84; P < 0.00001) (Figure 4G).

#### 3.3.5 Other indicators

This study involved the analysis of the number of GFAP-positive cells in the 3 included studies. There was significant heterogeneity in GFAP in the study (I^2^ = 78%, P = 0.004), which was analysed using a random-effects model. Compared with the control group, the number of GFAPs in the curcumin treatment group was significantly reduced (SMD = −1.72, 95% CI = −3.43∼-0.01; P = 0.05) (Figure 4H), although the p-value is only at the cut-off value of statistical differences, the possible reasons are considered to be the number of studies and the small sample size of the two groups.

**FIGURE 5:**
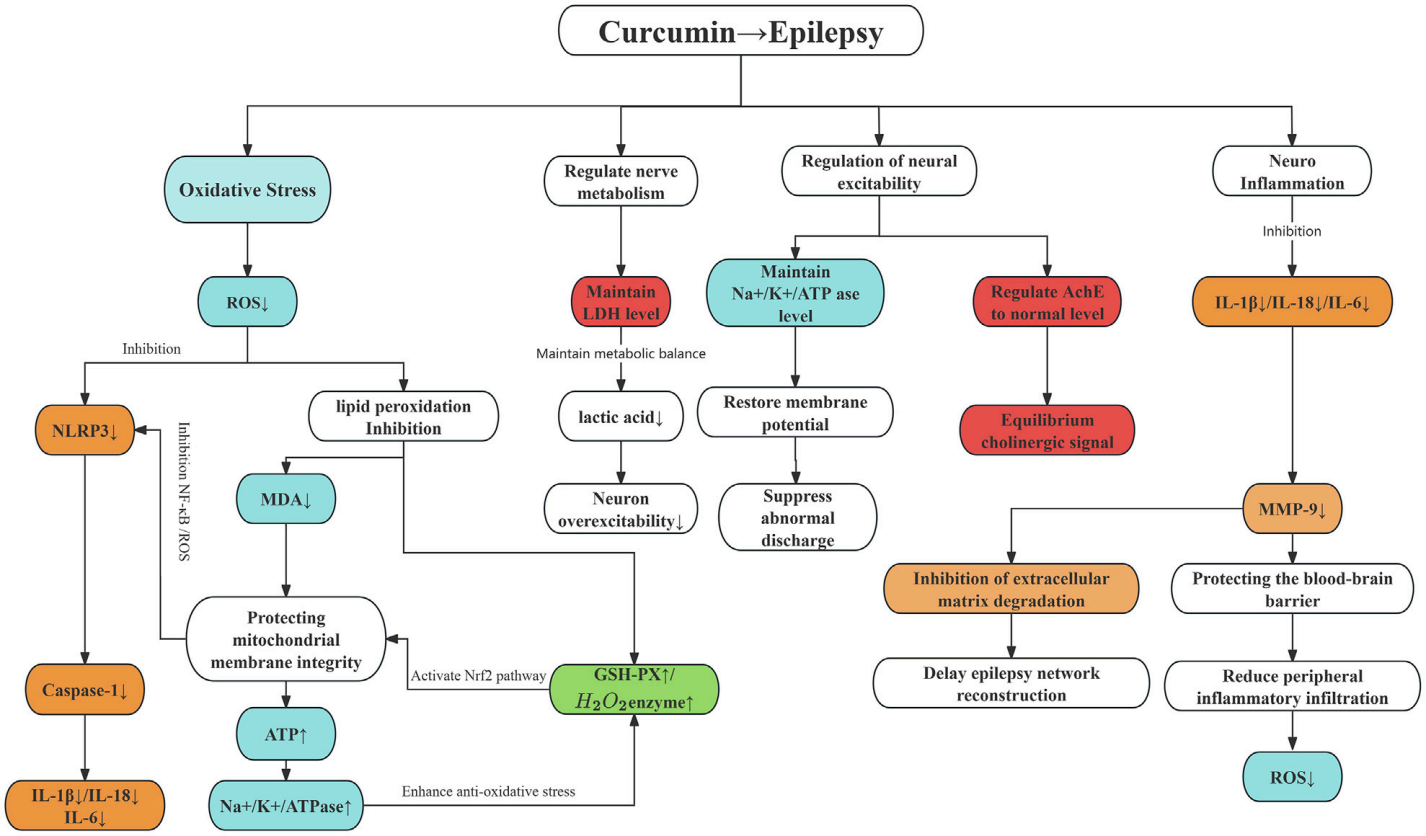
The mechanism of curcumin in treating epilepsy. Note: The colour area is the outcome index. Red represents the pro-epileptic pathway, green represents the antiepileptic pathway, blue represents oxidative stress, and orange represents inflammation.

In addition to all the above indicators, we found some outcome indicators can also be used to verify the treatment of epilepsy with curcumin. We summarized the mechanism of these indicators in treating epilepsy with curcumin, as shown in (Figure 5).

#### 3.3.6 Publication bias

**FIGURE 6:**
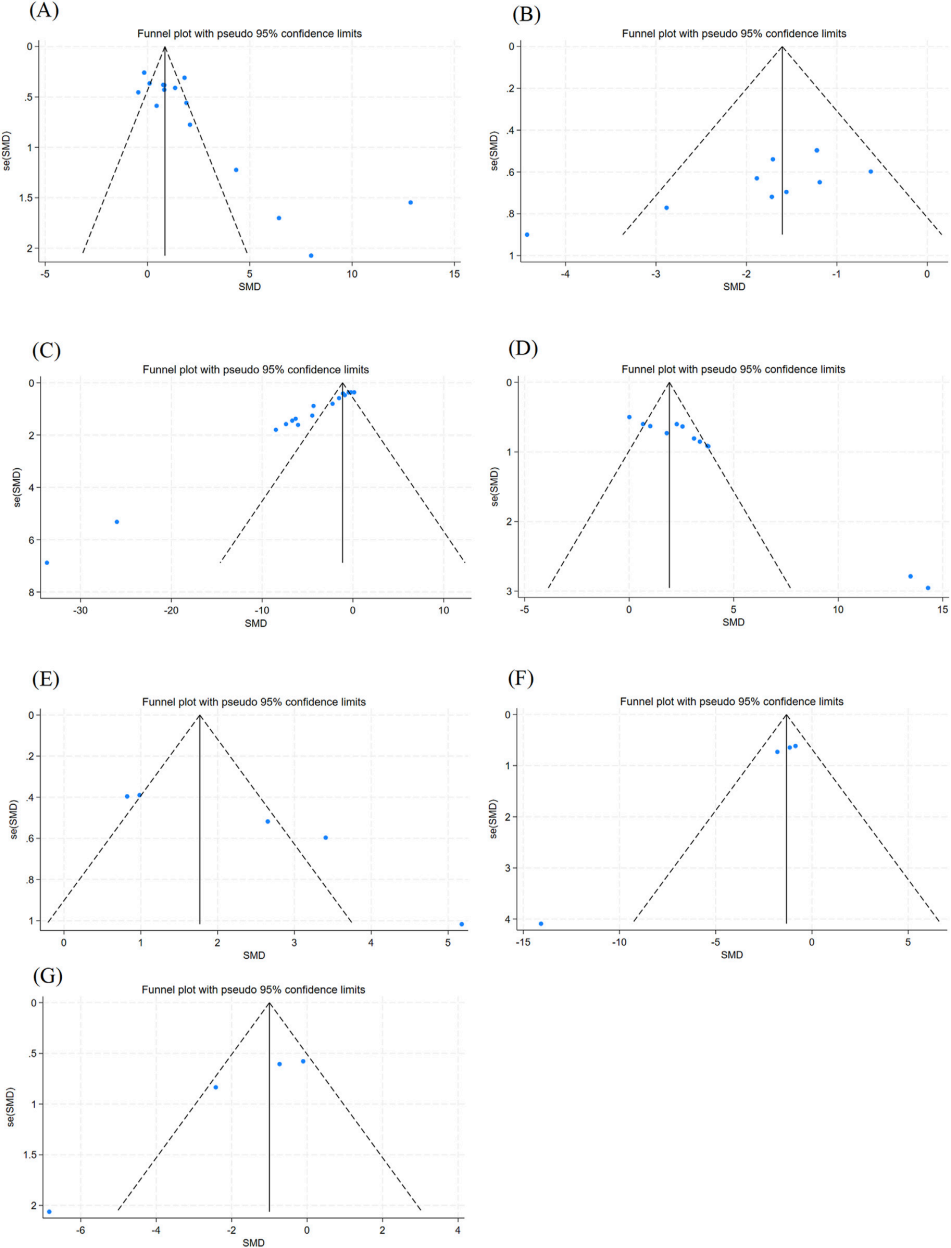
Outcome Index Funnel Plot. Note: **(A)** Epilepsy latency funnel plot; **(B)** Morris Water Maze escape latency funnel plot; **(C)** MDA content funnel plot; **(D)** GSH content funnel plot; **(E)** SOD content funnel plot; **(F)** IL-1β content funnel plot; **(G)** GFAP content funnel plot.

In this study, funnel plots were plotted for outcomes with more significant heterogeneity (Figure 6), and the Egger test (Table 2) was performed to rule out possible visual gaps and cases where the sample size was too small to be meaningful. It was found that there was obvious publication bias in the articles. It was considered that the literature included in this study was all animal experiments, and most of them were published with positive results.

**TABLE 2:** Egger test.

Number	Study	t	p	95% Conf. interval	
1	Epilepsy latency	3.98	0.002	2.259515	7.619399
2	MWM	−3.1	0.015	−9.538839	−1.404445
3	MDA	−17.66	0.000	−6.107179	−4.784479
4	GSH	7.41	0.000	4.350294	8.089155
5	SOD	4.63	0.019	2.539045	13.67035
6	IL-1β	−8.65	0.013	−5.741856	−1.927888
7	GFAP	−4.30	0.050	−9.595772	0.0073755

#### 3.3.7 Sensitivity analysis

**FIGURE 7:**
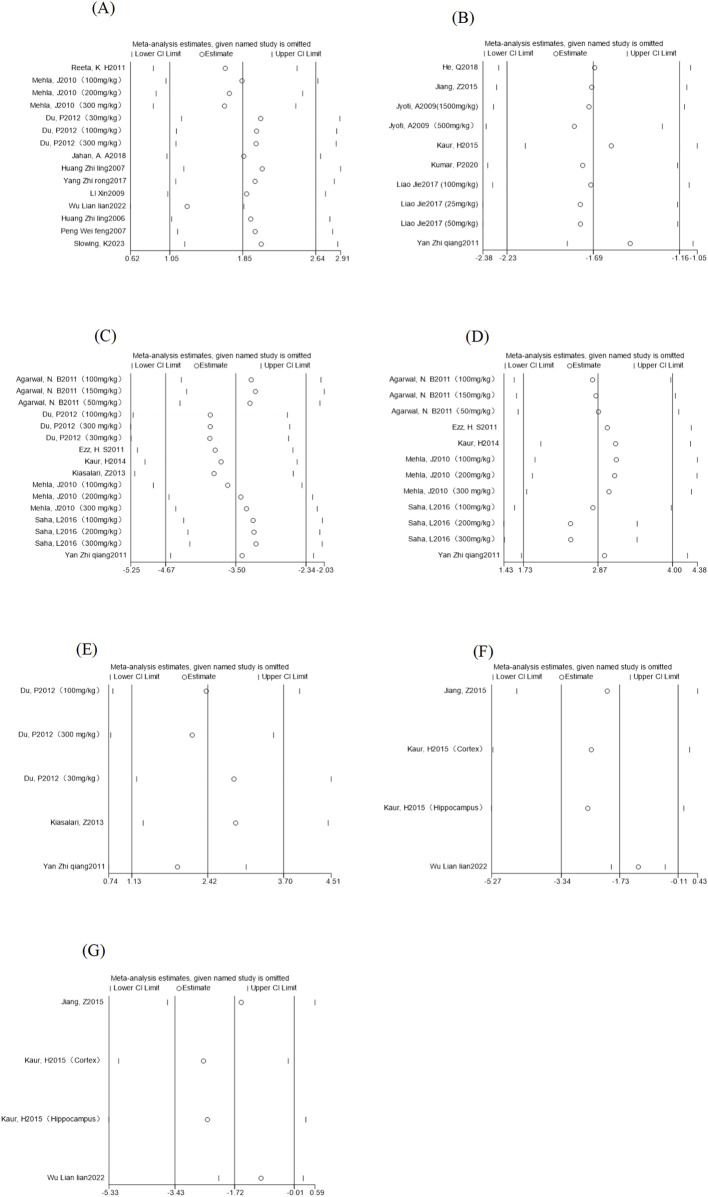
Outcome indicators sensitivity analysis. Note: **(A)** Epilepsy latency sensitivity analysis; **(B)** Morris Water Maze escape latency sensitivity analysis; **(C)** MDA content sensitivity analysis; **(D)** Sensitivity analysis of GSH content; **(E)** Sensitivity analysis of SOD content; **(F)** sensitivity analysis of IL-1β content; **(G)** Sensitivity analysis of GFAP content.

The pooled effect sizes from a new meta-analysis after a study was deleted were compared with the total response size to see if there was any change in the results. As shown in (Figure 7), there was no significant difference between the new pooled effect size and the total effect size for each outcome measure, and we believe that the above assessment results are still relatively stable and have corresponding significance.

## 4 Discussion

In 2010, the World Health Organization’s (WHO) Global Burden of Disease Study identified epilepsy as the second leading cause of neurological disability (Murray et al., 2012). As the global incidence of epilepsy continues to rise, more and more low- and middle-income countries are experiencing epilepsy, high preterm birth rates and mortality rates, and the psychosocial pressure faced by patients is increasing day by day. At present, the pathological mechanism of epilepsy has not been fully elucidated, and the existing theoretical systems cover many aspects, such as, oxidative stress, neuroinflammatory cascade, abnormal neural network remodelling, excitability-inhibition imbalance, ion channel dysfunction, abnormal glial cell proliferation, and disruption of blood-brain barrier integrity (Li et al., 2023; John, 2023). Oxidative stress and neuroinflammation, as the core pathological mechanisms of epilepsy research, have been highly concerned by academia in recent years.

Nowadays, a large amount of evidence shows that the brain is the organ with the highest metabolic demand in the human body, and epilepsy is one of the most common neurological diseases accompanied by oxidative stress. The hallmark of epilepsy is increased neuronal excitability, which means neuroinflammation and excessive production of reactive oxygen species (ROS) (Parsons et al., 2022). In terms of oxidative stress, previous studies have identified an increase in ROS production in epilepsy models, which is based on elevated markers of oxidative damage and redox homeostasis imbalance. In the Kainic Acid-induced temporal lobe epilepsy model, mitochondrial DNA oxidative damage and increased H_2_O_2_ levels have been observed (Jarrett et al., 2008).

Malondialdehyde (MDA) is genotoxic, carcinogenic and mutagenic, as the end product of lipid peroxidation and a key marker of the oxidative decomposition of polyunsaturated fatty acids. MDA disrupts cellular homeostasis and induces dysfunction by covalently modifying biological macromolecules (eg: DNA or proteins). Clinical studies have shown that MDA levels are characteristically elevated in oxidative stress-related diseases such as cardiovascular diseases, neurodegenerative diseases, and metabolic syndromes. Based on its formation mechanism and pathological association, MDA is widely recognized as a core biomarker that evaluates the degree of oxidative damage and the effect of antioxidant therapy (Dönmezdil et al., 2016).

Studies have shown that Glutathione (GSH) levels in the epilepsy lesions (hippocampus or cortex) are significantly reduced, accompanied by the accumulation of oxidative Glutathione (GSSG), suggesting the formation of a local oxidative micro environment (Cárdenas-Rodríguez et al., 2014; Pearson-Smith et al., 2017). As a key antioxidant of the central nervous system, the disorder of the GSH system may exacerbate the imbalance between oxidative damage and redox, leading to cognitive impairment and neuronal apoptosis. Lipid peroxidation and ferroptosis caused by loss of Glutathione peroxidase 4 (GPx4) have also been demonstrated in epilepsy models.

Major pathogenic agents such as kainate induce SE and significantly activate NOX2, an enzyme that generates superoxide anion (O^2-^) through a NADPH-dependent pathway, which is converted to H_2_O_2_ by disproportionation. Studies have shown that the over activation of NMDA receptors due to hyper glutamatergic states in epileptic states can significantly increase O^2-^ and H_2_O_2_ production through the NOX pathway (Brennan et al., 2009; Kovac et al., 2014). Notably, NOX activation alone triggers epileptiform discharge, and its inhibitors have shown significant antiepileptic effects in a variety of animal models (Malkov et al., 2019). Recent evidence suggests that the NOX2 isoenzyme, specifically expressed by microglia, may play a key regulatory role in ROS production after SE and glucose metabolism disorders during epilepticism (Fabisiak and Patel, 2022).

Studies have shown (Khayatan et al., 2022) that the immunoinflammatory response plays a key role in epilepsy-related brain injury, which induces neuronal overactivation and thus promotes seizures. Glial dysfunction is a common pathological feature of human and experimental models of epilepsy, which is manifested as the expression of microglial pro-inflammatory factors (IL-1β, IL-6, IL-17, TNF-α) in the early stage of epilepsy are significantly upregulated, resulting in impaired neuronal function. In addition, under the stimulation of inflammatory factors, microglia undergo structural remodelling to the pro-inflammatory phenotype, and astrocytes have an imbalance of energy homeostasis, which further promotes the neuronal overexcitation in the pathophysiological process of EP, resulting in neuronal damage and cell death in the brain, resulting in irreversible damage.

Curcumin, as a natural plant active ingredient, has shown an extraordinary place in developing epilepsy treatment. Several studies have found that it can reduce free radicals, helping to inhibit the activity of lipoxygenase (LOX), epoxygenase (COX), oxidized purine alcohol and nitric oxide synthase (NOS), thereby reducing the expression of free radicals under various pathophysiological conditions. *In vivo* and *in vitro* studies have shown that curcumin has a protective effect on some neurodegenerative diseases. Due to its important properties, such as anti-oxidation, anti-inflammation, anti-bacteria, anti-proliferation, anti-tumor, and anti-aging, it has been used to treat neurodegenerative diseases (Benameur et al., 2021). In addition, the combination of curcumin and antiepileptic drugs (AEDs) did not result in significant changes in serum levels of AEDs. These findings suggest that curcumin has considerable potential in the management of epilepsy as an adjunct to antiepileptic drugs, especially in minimizing dosage and side effects while improving efficacy (Wei et al., 2024).

During the screening and writing of this meta-analysis, we learned that curcumin can reduce the production of ROS, inhibit the inflammasome, reduce inflammatory factors, and protect the blood-brain barrier during seizures in treating epilepsy. It also inhibits the degradation of extracellular matrix, prevents neural network remodelling, and inhibits the occurrence of epilepticity, thereby reducing the production of ROS and preventing the secondary effect of oxidative stress on the brain in the brain. Curcumin can also affect the imbalance of energy metabolism, inhibit the increase of LDH content, reduce lactic acid accumulation, and prevent intracellular acidosis, thereby ensuring neuronal excitability. It can also affect the regulation of nerve excitability, maintain the balance of membrane potential, and restore the regular discharge of the brain. The intervention of curcumin reduces the production of oxidative stress, reduces the production of inflammatory factors, protects nerve cells, prevents apoptosis, maintains the normal production of Acetylcholinelinease (Ache), stabilizes synaptic transmission, further prevents the occurrence of epilepsy, and also slows down the progression of cognitive impairment in epilepsy patients.

In this meta-analysis, curcumin intervention doses were categorized into low (<100 mg/kg), moderate (100–299 mg/kg), and high (≥300 mg/kg) dose groups. Subgroup analysis showed that moderate and high dose groups demonstrated more consistent effects in prolonging seizure latency and improving oxidative stress and inflammatory indices. In contrast, the low dose group showed no significant differences due to insufficient sample size (only one study). Moderate and high-dose curcumin may more effectively activate the Nrf2 pathway, enhancing the activity of antioxidant enzymes (e.g.,SOD,GSH-Px), thereby inhibiting lipid peroxidation (significantly reduced MDA levels). Additionally, high-dose curcumin more potently inhibits nuclear factor kappa B (NF-κB), reducing microglial and astrocyte activation and decreasing the release of pro-inflammatory cytokines such as IL-1β and TNF-α. The lipophilic nature of curcumin enables it to more readily cross the blood-brain barrier at high doses, accumulating in epilepsy-related brain regions such as the hippocampus and cortex. Animal studies have shown that curcumin brain concentrations at 300 mg/kg can reach the effective threshold for inhibiting oxidative stress (approximately 10 μM) (Anand et al., 2007; Li et al., 2023). At the same time, low doses may exhibit unstable effects due to insufficient bioavailability. From the results of subgroup analysis, when the dose of curcumin was above 100 mg/kg/d, the therapeutic effect on epilepsy was positively correlated with the drug dose.

## 5 Conclusion

The results of this study revealed that curcumin significantly prolonged seizure latency and improved cognitive impairment compared with the control group. The literature analyzed also found that the natural compound also shortened the duration of status epilepticus (Jahan et al., 2018). In addition, this study systematically verified for the first time through quantitative analysis that curcumin has a precise dual mechanism of action against oxidative stress and inhibition of neuroinflammation in rodent epilepsy models. Although other potential benefit measures, such as neuroprotection in the hippocampus, were not included in this analysis due to the study design, the existing evidence has preliminarily suggested a multidimensional neurorepair potential.

However, there are some limitations in this meta-analysis, including quality assessment, there is an increased risk of detection and selection bias, as many included studies did not report details of blinding implementation and random allocation concealment. Significant heterogeneity exists in some outcome indicators, and although subgroup analysis explored the dose-effect relationship, differences in model types (such as PTZ and pilocarpine modeling), administration regimens, and detection methods between studies may still affect the consistency of results. The number of studies on low-dose curcumin is insufficient, limiting the efficacy of subgroup analysis, and more high-quality studies are needed to verify its specific efficacy and safety. Methodologically, issues like indicator heterogeneity, lack of original data in some studies, potential language bias (only including English and Chinese literature), bias in data extraction, and publication bias toward positive results may impact the conclusions. More importantly, there are noticeable species differences between rodents and humans, which makes it difficult for existing animal models to simulate the complex pathological features of human epilepsy, which challenges the effectiveness of translating preclinical research conclusions into clinical practice.

Although this study confirms that curcumin improves the seizure and cognitive function of epileptic rodent models through antioxidant and anti-inflammatory mechanisms, its clinical application still needs to break through species differences and methodological limitations. In the future, high-quality clinical studies are needed to verify its safety and effectiveness, especially focusing on the potential of adjuvant therapy for patients with drug-resistant epilepsy.

## Data Availability

The original contributions presented in the study are included in the article/supplementary material, further inquiries can be directed to the corresponding author.
